# Psychometric Properties of the COVID-19 Pandemic Fatigue Scale: Cross-sectional Online Survey Study

**DOI:** 10.2196/34675

**Published:** 2022-09-08

**Authors:** Carmen Rodriguez-Blazquez, Maria Romay-Barja, Maria Falcon, Alba Ayala, Maria João Forjaz

**Affiliations:** 1 National Epidemiology Center Carlos III Health Institute Madrid Spain; 2 Nacional Center of Tropical Diseases Carlos III Health Institute Madrid Spain; 3 School of Medicine University of Murcia Murcia Spain; 4 Department of Statistics Carlos III University Getafe Spain

**Keywords:** COVID-19, pandemic fatigue, psychometric properties, Rasch analysis, validation, online survey, pandemic, fatigue, mental health, information seeking, health information

## Abstract

**Background:**

Pandemic fatigue is defined as feelings of demotivation to follow preventive measures against COVID-19, together with decreased trust in government and frequency of information-seeking behaviors.

**Objective:**

This study aims to analyze the psychometric properties of the COVID-19–specific pandemic fatigue scale according to classical test theory (CTT) and Rasch model approaches in the general Spanish population.

**Methods:**

This was a cross-sectional study in a representative sample of 1018 adults who completed an online survey in November 2020 in the framework of the COVID-19 Snapshot Monitoring (COSMO)-Spain project. The assessments included the 6-item COVID-19 Pandemic Fatigue Scale (CPFS) and other COVID-19–related variables: COVID-19 infection, adherence to preventive behaviors, information-seeking behavior, self-efficacy, worry, and cognitive and affective risk perception. Data quality, acceptability, reliability, and validity were analyzed according to CTT, and the fit to the Rasch model, unidimensionality, appropriateness of the response scale, item local independency, reliability (person-separation index [PSI]), and item-person distribution were also calculated.

**Results:**

The mean CPFS score was 17.06 (SD 5.04, range 6-30), with higher scores for women, younger participants, participants who never seek information on COVID-19, those who think they would contract a mild disease in case of infection, those with higher level of worry about coronavirus/COVID-19, and those who felt depressed or felt the coronavirus/COVID-19 is spreading slowly (all *P*<.01). The Cronbach alpha for the CPFS was 0.74. In the confirmatory factor analysis, one factor was identified (root mean square error of approximation [RMSEA]=.02; comparative fit index [CFI]=.99; χ^2^_5_=8.06, *P*=.15). The CPFS showed good fit to the Rasch model (χ ^2^_24_=42.025, *P*=.01, PSI=.642), unidimensionality (binomial 95% CI –.005 to .045), and item local independency.

**Conclusions:**

Our results suggest that the CPFS has moderate reliability and internal consistency and it is composed of a single dimension. It is a useful tool to ascertain the level of pandemic fatigue in the general population, which may help to guide the communication and information strategies to face the COVID-19 pandemic.

## Introduction

The world has become familiar with the term “pandemic fatigue” in the context of COVID-19 [1-3]. This term has been used to describe different phenomena related to psychological distress and demotivation to follow preventive measures, as well as decreased trust in the government and frequency of information-seeking behaviors [3].

In 2020, the World Health Organization (WHO) defined pandemic fatigue as a “demotivation to follow recommended protective behaviors, emerging gradually over time and affected by a number of emotions, experiences and perceptions,” proposing a framework on how to “maintain and reinvigorate” people’s motivation to comply with COVID-19 response policies [4]. The WHO proposes that pandemic fatigue is expressed through an increasing number of people not sufficiently following or accepting recommendations and restrictions or decreasing their effort to keep themselves informed about the pandemic [4].

The restrictions adopted by the authorities to tackle this public health crisis have saved many lives but have also affected the mental and physical well-being of the population, social cohesion, economic stability, and community resilience [5]. After more than 2 years of restrictions, fatigue is an expected and natural response [4], and several authors have already measured how support of and compliance with nonpharmaceutical interventions (NPIs) have decreased in Spain [6] and worldwide as the pandemic evolves [7-12].

In Spain, compliance with health authorities’ recommendations has been high [13], and the vaccination campaign has been a success [14,15]. Even so, compliance with NPIs remains important and will be in the future, as long as new variants continue to be a threat [16]. On the other hand, social, psychological, and economic consequences of the pandemic will continue over time, so the availability of a tool that measures pandemic fatigue is crucial.

Another challenge that public health authorities face is to keep the population informed in the context of a health crisis of unpredictable duration. Disinterest and information fatigue might be an obstacle for adherence to NPIs to combat the pandemic [17]. In Spain, there is a national strategy to improve health communication and address pandemic fatigue [18]. Public health communication strategies should focus on raising awareness in the event of future outbreaks and new restrictions [17].

Lilleholt et al [19], in 2021, conceptualized pandemic fatigue to represent a general demotivation toward following COVID-19–related health protective behaviors and staying informed about the development of the pandemic. The authors developed and validated the COVID-19 Pandemic Fatigue Scale (CPFS) with the aim of identifying who experiences it, analyzing related emotions and perceptions, and shedding light on the relationship between pandemic fatigue and health protective behaviors.

The aim of this study was to assess the psychometric properties of the Spanish version of the CPFS to measure pandemic fatigue in the Spanish general population, administered online, using 2 complementary methodological approaches: classic test theory (CTT) and the Rasch model. In addition to reliability and internal validity, Rasch analysis provides unique information such as differential item functioning for population groups and adequacy of the response scale. Finally, scales that fit the Rasch model provide results in a linear scale.

## Methods

### Design and Procedures

This is a cross-sectional, observational, nationwide study with survey data collected using an online questionnaire. This study is part of a larger project, the COVID-19 Snapshot Monitoring (COSMO)-SPAIN project [20], based on the COSMO tool developed by the WHO Europe Regional Office [6,21], with the aim of monitoring the knowledge, attitudes, compliance with the preventive measures, and risk perception of the Spanish population toward the COVID-19 pandemic, as well as informing COVID-19 outbreak response measures, including policies, interventions, and communications. More details can be found in the protocol of the COSMO-Spain study [22].

A nationally representative sample of 1018 subjects living in Spain was recruited. The sample was stratified to match the Spanish general population in terms of age, education, gender, and area of residence. A research company invited the potential participants who fit the inclusion criteria (both sexes, aged 18 years or older, and being able to answer an online questionnaire) and carried out the survey. The research market company has a panel of 157,535 members from the Spanish population. They contacted panel members who fit the inclusion criteria by email; 2655 invitations were sent, 1777 members participated in the survey (response rate 67%), and 1020 complete questionnaires were obtained. The data were collected between November 24, 2020, and November 27, 2020, at the end of the “second pandemic wave” in Spain. During that period, 60,462 cases of COVID-19 were detected, with a cumulative incidence of 128.6 over 14 days [23]. Mobility restrictions and capacity limitations in commercial establishments were present in different Spanish regions.

### Ethical Review

The Ethics Committee of Carlos III Health Institute (CEI PI 59-2020-v2) approved the study protocol. The survey was anonymous, and the research company provided data with no identifying information to the researchers. Participants were informed on the purpose and characteristics of the study and provided informed consent by clicking a box.

### Variables

#### Online Survey

The online survey included questions about participants’ sociodemographic characteristics: sex (male, female), age, education (highest level of education attained: incomplete primary or less, primary, secondary, high school, and university), area of residence (village, 2000 to 50,000; town, 50,000 to 400,000; city, >400,000), and employment situation (working, student, domestic care, retired/pensioner, long-term unemployed, unemployed, or Spanish temporary employment regulation due to COVID-19).

##### CPFS

The CPFS is a self-reported questionnaire based on the original version by Lilleholt et al [19]. It asks about demotivation toward COVID-19–related health-protective behaviors and staying informed about the development of the pandemic. The CPFS includes 6 items rated from 1 (strongly disagree) to 5 (strongly agree): “I am tired of all the COVID-19 discussions in TV shows, newspapers and radio programs, etc.,” “I feel strained from following all of the behavioral regulations and recommendations around COVID-19,” “I am sick of hearing about COVID-19,” “I am tired of restraining myself to save those who are most vulnerable to COVID-19,” “when friends or family members talk about COVID-19, I try to change the subject because I do not want to talk about it anymore,” and “I am losing my spirit to fight against COVID-19.” The total CPFS score is obtained by summing the items, with a maximum of 30 points that is indicative of a higher degree of COVID-19 pandemic fatigue. In the original study, it reached Cronbach α values of 0.83 and 0.87 (Danish and German studies, respectively) [19].

##### Other Variables

Other variables were included, as described in the following paragraphs (see also Multimedia Appendix 1).

COVID-19 infection was ascertained using the question “To your knowledge, are you, or have you been, infected with COVID-19?”, with yes/no response options.

Adherence to preventive behaviors was assessed by asking how frequently respondents carried out a list of 12 measures to prevent infection from coronavirus/COVID-19, with 1 (never) to 5 (always) as scoring options. The listed behaviors included use of face masks: (1) using face masks following the recommendations and (2) wearing face masks in the presence of relatives and friends. It also included questions on hygienic behavior: (3) ventilating closed spaces; (4) using hydro alcoholic gel or disinfectants; (5) disinfecting surfaces; (6) washing hands; and (7) avoiding touching eyes, nose, and mouth with unwashed hands. Finally, it included physical distancing: (8) avoiding public transportation, (9) ensuring physical distancing, (10) avoiding social or family events, (11) not visiting relatives and friends if they are in quarantine, and (12) avoiding crowded spaces. The total number of preventive behaviors was calculated for each participant, computing the scores 4 and 5 as a positive response (=1) and summing them to obtain a score ranging from 0 to 12. The same procedure was applied to calculate the score for each type of preventive behavior.

Information-seeking behavior was assessed by asking respondents about the frequency of searching for information on coronavirus/COVID-19, answered on a scale from 1 (never) to 5 (several times a day).

Perceived self-efficacy was surveyed using the question “avoiding an infection with coronavirus/COVID-19 in the current situation is...?”, with a response scale from 1 (very difficult) to 5 (very easy). This question, addressing self-assessed COVID-19 self-protection and avoidance ability, has been adapted from a previous study [24] by the original authors of the COSMO survey [21].

Level of worry about the coronavirus/COVID-19 in general was collected using a response scale from 1 (do not worry at all) to 5 (worry a lot).

Cognitive risk perception was measured using a question on perceived probability of getting infected with coronavirus/COVID-19, answered from 1 (very unlikely) to 5 (very likely), and a question on how severe would contracting the coronavirus/COVID-19 be for you, answered on a scale from 1 (not severe) to 5 (very severe) [19]. Both items were multiplied to obtain the value of the cognitive risk perception, ranging from 1 to 25, with higher scores indicative of higher risk perception.

Affective risk perception was collected using “the coronavirus/COVID-19 to me feels...,” including 3 items: speed of propagation, with a response scale ranging from 1 (spreading slowly) and 5 (spreading fast); fear, scored from 1 (not fear-inducing) to 5 (fear-inducing); and mood, with a scale from 1 (it does not affect my mood) to 5 (makes me feel depressed) [19]. The responses to these questions were summed, obtaining a score that ranged from 3 to 15, with higher values indicating higher affective risk perception.

All items were originally in English and were translated by professional translators, reviewed and slightly modified by the COSMO-Spain team to adapt them to the Spanish context.

### Data Analysis

Variables were summarized using descriptive statistics, including central tendency and dispersion measures (mean, median, and SD) and frequency and percentages, depending on their format.

Since the total CPFS score fit a normal distribution (Shapiro-Wilk test, *P*=.26), parametric statistics were used. According to the CTT [25], the following psychometric properties of the CPFS were analyzed: data quality and acceptability, structural validity, hypotheses testing (construct validity), and internal consistency.

Data quality and acceptability were computed by the mean, median, SD, and range of the observed versus theoretical values; skewness (criterion: −1 to +1); floor and ceiling effects (criterion: ≤15%) of the CPFS items; and total score [26].

For structural validity, exploratory (EFA) and confirmatory factor analyses (CFA) were used. For EFA, a principal component analysis with varimax rotation was applied. CFA used maximum likelihood estimations. A root mean squared error of approximation (RMSEA) ≤0.06 and comparative fit index (CFI) >0.9 indicated a good fit to the model [27].

Hypotheses testing (construct validity) included convergent and discriminative validity. Convergent validity was analyzed using Pearson correlation coefficients to ascertain the association of pandemic fatigue with related continuous variables: age, number and type of protective behaviors, and cognitive and affective risk perception. Following the literature [4,19], moderate-to-high correlation coefficients (*r*≥0.30 and *r*≥0.60) [28] between CPFS and these variables were hypothesized. Regarding discriminative (known groups) validity, mean differences in total CPFS score in the sample grouped by relevant variables were calculated, using ANOVA and Student *t* tests. The following hypotheses were established, according to the literature [4,12]: a higher CPFS score would be reached in younger participants; those with lower education levels, risk perception, perceived severity, self-efficacy, level of worry, or information-seeking behavior; and those with higher levels of depression.

Internal consistency was examined by computing the Cronbach α coefficient (criterion ≥0.70), item total corrected correlations (standard *r* ≥0.40), interitem correlations, and the item homogeneity index (criterion >.30) [29].

The Rasch model, one of the most used applications of item response theory, was also applied to complete the information on the measurement properties of the CPFS provided by the CTT. According to the Rasch model, the answer to a certain item is a function between the person’s ability (level of pandemic fatigue) and the item’s difficulty (level of construct represented by that item), expressed in logits [30]. The following measurement properties were assessed: fit to the Rasch model, unidimensionality, appropriateness of the response scale, item local independency, reliability (person-separation index [PSI]), and item-person distribution. There are excellent tutorials and examples explaining the Rasch analysis process [31,32].

Since small deviations from the Rasch model are signaled as statistically significant when using large sample sizes, resulting in unnecessary model modifications, a random sample of 300 was drawn. This sample size allows for stable estimates regardless of targeting [33]. Fit to the Rasch model was considered when there was a nonsignificant chi-square test using Bonferroni correction for number of items (*P*>.008) [31]. Also, fit residuals were expected to be within the interval of –2.5 to +2.5 and item and person estimates to follow a normal distribution with a mean of 0 and SD of 1. Modifications were performed iteratively until model fit is achieved. PSI measures reliability and is interpreted similarly to Cronbach alpha. Threshold is the point of equal answer probability between 2 adjacent response categories. In case of disordered thresholds, adjacent response categories were collapsed. Unidimensionality was checked using a principal component analysis of residuals and then comparing person estimates with a binomial test; a lower bound of the 95% CI should be ≤.05 [34,35]. Local item independency, or the degree to which 1 item response does not lead to the response to another item, was analyzed though the correlation matrix of the residuals [36]. Differential item functioning (DIF) occurs when, for the same construct level, 2 or more sample groups answer in a statistically different way [37]. DIF was inspected through ANOVA by the following groups: age (groups defined by the median: ≤46 years; >46 years), gender, and education level (low: up to 14 years old; medium: secondary or professional training; high: university). Finally, the person-item threshold distribution was visually inspected.

CTT analysis was performed using SPSS 27.0 (IBM Corp, Armonk, NY) and Rasch analysis using RUMM2030 statistical software.

## Results

The sample was formed by the same number of men and women, with a mean age 46.1 (SD 14.2, range 18-85) years (Table 1). Most participants were working (577/1018, 56.7%), 27.7% (282/1018) of them in a setting with a moderate risk of infection.

**Table 1:** Sociodemographic characteristics of the sample (n=1018) in the COVID-19 Snapshot Monitoring (COSMO)-Spain study, November 2020.

Variables		Results, n (%)
**Sex**		
	Women	509 (50.0)
	Men	509 (50.0)
**Age groups (years)**		
	18-29	177 (17.4)
	30-44	301 (29.6)
	45-60	336 (33.0)
	≥61	204 (20.0)
**Education level**		
	Incomplete primary or less	31 (3.0)
	Primary	240 (23.6)
	Secondary	308 (30.3)
	University	439 (43.1)
**Employment**		
	Working	577 (56.7)
	Student	41 (4.0)
	Homemaker	32 (3.1)
	Retired/pensioner	186 (18.3)
	Long-term unemployed	100 (9.8)
	Unemployed or ERTE^a^	82 (8.1)
**Type of work**		
	With high risk of contagion	101 (9.9)
	With moderate risk of contagion	282 (27.7)
	No risk	69 (6.8)
	Telework	102 (10.0)
	Health care staff	23 (2.3)

^a^ERTE: Spanish Temporary Employment Regulation due to COVID-19; in Spanish, “expediente de regulación temporal de empleo.”

### Psychometric Properties According to CTT

Table 2 shows the data quality and acceptability analysis of the CPFS. The mean total CPFS score was 17.06 (median 17.0, SD 5.04, range 6-30). Skewness of the total CPFS score was .13. All items reached the expected score range (1-5), and most of them showed a floor effect, especially items 6 and 4. Ceiling effect was marked in items 1 and 3. The total PFS score did not present floor or ceiling effects.

**Table 2:** Data quality and acceptability of the COVID-19 Pandemic Fatigue Scale (CPFS) in the COVID-19 Snapshot Monitoring (COSMO)-Spain study, November 2020.

CPFS item	Mean (SD)	Median	Minimum to maximum	Floor effect, %	Ceiling effect, %
1. I am tired of all the COVID-19 discussions in TV shows, newspapers and radio programs, etc.	3.88 (1.23)	4.00	1-5	7.3	43.3
2. I feel strained from following all of the behavioral regulations and recommendations around COVID-19	2.77 (1.32)	3.00	1-5	24.2	12.5
3. I am sick of hearing about COVID-19	3.52 (1.28)	4.00	1-5	9.8	30.3
4. I am tired of restraining myself to save those who are most vulnerable to COVID-19	2.07 (1.27)	2.00	1-5	48.3	7.2
5. When friends or family members talk about COVID-19, I try to change the subject because I do not want to talk about it anymore	2.81 (1.30)	3.00	1-5	21.6	13.5
6. I am losing my spirit to fight against COVID-19	2.01 (1.20)	2.00	1-5	49.7	5.1
CPFS Total	17.06 (5.04)	17.00	6-30	1.9	1.4

**Figure 1:**
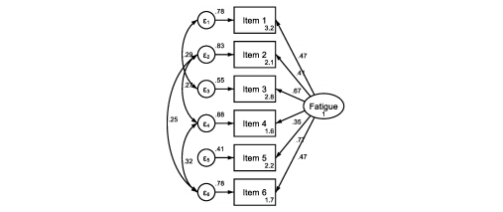
Path diagram of the COVID-19 Pandemic Fatigue Scale (CPFS) model in the COVID-19 Snapshot Monitoring (COSMO)-Spain study, November 2020. CPFS items: 1. I am tired of all the COVID-19 discussions in TV shows, newspapers and radio programs, etc.; 2. I feel strained from following all of the behavioral regulations and recommendations around COVID-19; 3. I am sick of hearing about COVID-19; 4. I am tired of restraining myself to save those who are most vulnerable to COVID-19; 5. When friends or family members talk about COVID-19, I try to change the subject because I do not want to talk about it anymore; 6. I am losing my spirit to fight against COVID-19.

Regarding structural validity, EFA identified 2 factors with a correlation coefficient of .70, explaining 62.6% of variance. Figure 1 shows the path diagram of the CPFS using CFA. The 1-factor model obtained an RMSEA of .02 and CFI of .99 (χ^2^_5_=8.06, *P*=.15).

The construct validity results of the CPFS appear in Table 3. CPFS scores were significantly higher for women, younger participants, participants who never seek information on COVID-19, those who think they would contract a mild disease in case of infection, those with lower level of worry about coronavirus/COVID-19, those who felt depressed, or participants who felt the coronavirus/COVID-19 was spreading slowly. The CPFS correlated with age (*r*=–0.20; *P*<.001), number of preventive measures (*r*=–0.16; *P*<.001), use of face masks and physical distancing (*r*=–0.12; *P*<.001), hygienic behavior (*r*=–0.13; *P*<.001), and affective risk perception (*r*=0.07; *P*=.03).

Internal consistency statistics are displayed in Table 4. The Cronbach α was .74, item homogeneity was .33, and item-total corrected correlation ranged from *r*=0.42 (item 1) to *r*=0.56 (item 3). Intercorrelation between items ranged from *r*=0.17 (item 1 with items 4 and 6) to *r*=0.51 (item 1 with items 3 and 5).

**Table 3:** Construct validity of the COVID-19 Pandemic Fatigue Scale (CPFS) in the COVID-19 Snapshot Monitoring (COSMO)-Spain study, November 2020.

Variables		Number of participants, n	CPFS scores, mean (SD)	*P* value^a^	Correlation with total CPFS^b^	*P* value
**Sex**						
	Men	509	16.59 (4.75)	.003	—^c^	—
	Women	509	17.53 (5.28)		—	—
Age (years)^d^		—	—	—	–0.20	<.001
**Age group (years)**						
	18-29	177	18.90 (5.07)	<.001	—	—
	30-44	301	17.32 (4.93)		—	—
	45-60	336	16.51 (5.21)		—	—
	≥61	204	15.99 (4.42)		—	—
**Education level**						
	Primary or less	78	17.67 (5.53)	.47	—	—
	Secondary	501	16.92 (4.97)		—	—
	University	439	17.10 (5.04)		—	—
**COVID-19 infection (past or present)**						
	Yes	69	17.45 (4.72)	.40	—	—
	No	949	17.03 (5.07)		—	—
Number of preventive behaviors^d^		—	—	—	–0.16	<.001
**Type of preventive behaviors**						
	Use of face masks^d^	—	—	—	–0.12	<.001
	Hygienic behavior^d^	—	—	—	–0.13	<.001
	Physical distancing^d^	—	—	—	–0.12	<.001
**Information-seeking behavior**						
	Never (1-2)	270	18.53 (5.08)	<.001	—	—
	Occasionally (3)	402	16.67 (4.45)		—	—
	Several times a day (4-5)	346	16.35 (5.43)		—	—
**Perceived self-efficacy: for me, avoiding an infection with coronavirus/COVID-19 is...?**						
	Very difficult/difficult (1-2)	246	17.52 (5.22)	.19	—	—
	Neutral (3)	528	17.01 (4.72)		—	—
	Easy/very easy (4-5)	244	16.70 (5.50)		—	—
**General level of worry**						
	Not worry at all (1-2)	116	18.81 (5.56)	<.001	—	—
	Moderate (3)	301	17.55 (4.66)		—	—
	Worry a lot (4-5)	601	16.47 (5.02)		—	—
Cognitive risk perception^d^		—	—	—	–0.01	.85
**Perceived probability of getting infected with coronavirus/COVID-19**						
	Very unlikely/unlikely (1-2)	268	17.24 (5.29)	.03	—	—
	Neutral (3)	487	16.65 (4.63)		—	—
	Likely/very likely (4-5)	263	17.62 (5.46)		—	—
**How severe would contracting the coronavirus/ COVID-19 be for you**						
	Very mild/mild (1-2)	176	17.59 (5.67)	.03	—	—
	Normal (3)	480	17.28 (4.50)		—	—
	Severe/very severe (4-5)	362	16.51 (5.35)		—	—
Affective risk perception^d^		—	—	—	0.07	.03
**The coronavirus/COVID-19 to me feels…**						
	It is spreading slowly (1-2)	31	19.58 (6.37)	.01	—	—
	Neutral (3)	185	17.36 (4.55)		—	—
	It is spreading fast (4-5)	802	16.89 (5.07)		—	—
**The coronavirus/ COVID-19 to me feels…**						
	Not fear-inducing (1-2)	265	17.02 (5.40)	.96	—	—
	Neutral (3)	344	17.12 (4.61)		—	—
	Fear-inducing (4-5)	409	17.03 (5.16)		—	—
**The coronavirus/ COVID-19 to me feels…**						
	It does not affect my mood (1-2)	275	15.97 (4.94)	<.001	—	—
	Neutral (3)	319	16.76 (4.60)		—	—
	Depressed (4-5)	424	17.99 (5.26)		—	—

^a^Student *t* and ANOVA tests with Bonferroni correction.

^b^Pearson correlation coefficients for continuous variables.

^c^Not applicable.

^d^Continuous variable.

**Table 4:** Internal consistency of the COVID-19 Pandemic Fatigue Scale (CPFS) in the COVID-19 Snapshot Monitoring (COSMO)-Spain study, November 2020.

CPFS item	ITCC^a^	Cronbach α after item deletion	Intercorrelations					
			Item 1	Item 2	Item 3	Item 4	Item 5	Item 6
1. I am tired of all the COVID-19 discussions in TV shows, newspapers and radio programs, etc.	.42	.73	—^b^	0.21	0.51	0.17	0.37	0.17
2. I feel strained from following all of the behavioral regulations and recommendations around COVID-19	.46	.72	0.21	—	0.29	0.37	0.30	0.39
3. I am sick of hearing about COVID-19	.56	.69	0.51	0.29	—	0.23	0.51	0.31
4. I am tired of restraining myself to save those who are most vulnerable to COVID-19	.42	.72	0.17	0.37	0.23	—	0.26	0.43
5. When friends or family members talk about COVID-19, I try to change the subject because I do not want to talk about it anymore	.54	.69	0.37	0.30	0.51	0.26	—	0.37
6. I am losing my spirit to fight against COVID-19	.50	.70	0.17	0.39	0.31	0.43	0.37	—

^a^ITCC: item total corrected correlation.

^b^Not applicable.

### Psychometric Properties According to the Rasch Model

The Rasch analysis showed that all items displayed disordered thresholds. After reducing the response options to scales with 2 to 4 points, according to the item, data showed a good fit to the Rasch model (χ ^2^_24_=42.025; *P*=.01; PSI=.642; Table 5), unidimensionality (binomial 95% CI: –005 to .045), and item local independency.

**Table 5:** Goodness of fit to the Rasch Model of the COVID-19 Pandemic Fatigue Scale (CPFS) in the COVID-19 Snapshot Monitoring (COSMO)-Spain study, November 2020.

Attribute		Criteria	CPFS
**Item fit residual**			
	Mean	0	–.189
	SD	1	.852
**Person fit residual**			
	Mean	0	–.285
	SD	1	.736
Item-trait, χ^2^ (df)		Low	42.025 (24)
Interaction *P* value		NS^a^	.0128
PSI^b^		>0.70	.642
**Unidimensionality**			
	Independent *t* tests	<5%	2.00%
	95% CI binomial	*^c^	.042-.091

^a^NS: nonsignificant.

^b^PSI: Personal Separation Index

^c^Lower bound should be ≤.05.

**Figure 2:**
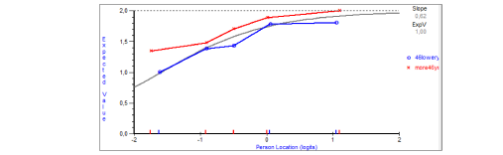
Differential item functioning for item 1, by age groups defined by the median (≤46 years, >46 years) in the COVID-19 Snapshot Monitoring (COSMO)-Spain study, November 2020.

**Figure 3:**
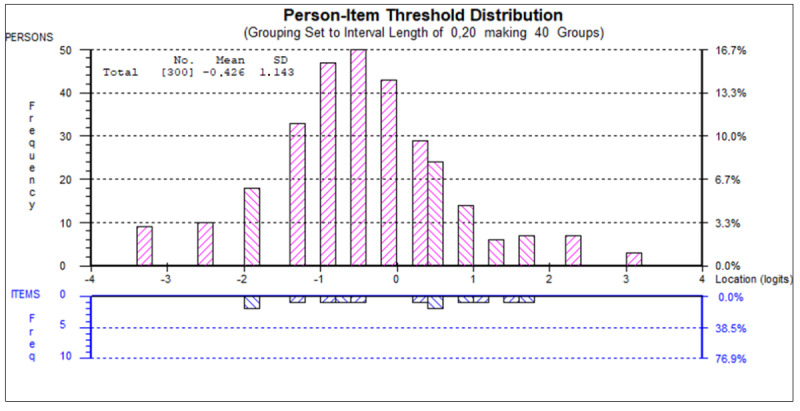
Person-item threshold distribution of the COVID-19 Pandemic Fatigue Scale (CPFS) in the COVID-19 Snapshot Monitoring (COSMO)-Spain study, November 2020. Note: the distribution of persons (top part) and item thresholds (bottom part) locations are shown on the same logit scale. Persons with higher fatigue level and more difficult thresholds are represented on the right.

Table 6 presents the fit at the item level. Item 1 (“I am tired of all the COVID-19 discussions in TV shows, newspapers and radio programs, etc.”) showed DIF by age, with older adults overestimating pandemic fatigue (Figure 2). No DIF was observed by sex or education level. The person-item threshold distribution was close to normality, with no floor or ceiling effects and item threshold locations ranging from –2 to 2 logits. There was a lack of items representing persons with lower and higher pandemic fatigue levels (Figure 3).

**Table 6:** Individual item fit of the COVID-19 Pandemic Fatigue Scale (CPFS) in the COVID-19 Snapshot Monitoring (COSMO)-Spain study, November 2020.

Item	Location	Standard error	Fit residual	χ^2^_4_	*P* value
1. I am tired of all the COVID-19 discussions in TV shows, newspapers and radio programs, etc.	–1.596	.099	1.177	8.672	.07
2. I feel strained from following all of the behavioral regulations and recommendations around COVID-19	0.289	.074	0.029	5.762	.22
3. I am sick of hearing about COVID-19	–1.346	.097	–1.179	10.429	.03
4. I am tired of restraining myself to save those who are most vulnerable to COVID-19	1.777	.183	–0.766	7.832	.10
5. When friends or family members talk about COVID-19, I try to change the subject because I do not want to talk about it anymore	–0.220	.090	0.251	3.163	.53
6. I am losing my spirit to fight against COVID-19	1.095	.088	–0.647	6.167	.19

## Discussion

Pandemic fatigue is an important barrier to implementing NPIs, and monitoring its population levels requires the availability of valid and reliable measures [4]. This is the first validation study of the Spanish version of the CPFS scale in a representative sample of the Spanish general population, as part of an international behavioral insights initiative, the COSMO study [6,21]. The use of 2 complementary methodological approaches, the CTT and Rasch model, provides a robust testing of the psychometric properties of the scale.

### Principal Findings

Results indicate that the Spanish population reported a moderate-to-high level of pandemic fatigue during the second pandemic wave (mean 17.06 points on a 24-point scale). Our data are in accordance with those of another Spanish study that reported a moderate level of pandemic fatigue (around 3 on a 1-4 scale) in the same period, using a different scale [38].

The CPFS displayed moderate reliability and internal consistency, allowing for group comparisons. Both CFA and Rasch analysis supported that the scale measures a single construct, indicating that the items may be summed to provide a meaningful total score. In addition, Rasch analysis allows converting the raw scores into a true interval scale, supporting the calculation of change scores and use of parametric statistics. The Rasch analysis results also indicate that respondents were not able to distinguish between all levels of the 5-point response scale. If these results are confirmed in further studies, the response scale may be simplified. However, there is no need to change the way the scale is administered, only how it is coded.

Items 1 (I am tired of all the COVID-19 discussions in TV shows, newspapers and radio programs, etc.) and 3 (I am sick of hearing about COVID-19) had a ceiling effect, indicating that a high percentage of respondents scored the highest level. This is consistent with the findings from the Rasch analysis, where these items presented the lowest locations, meaning that people with low levels of pandemic fatigue will easily endorse items 1 and 3. On the contrary, items 4 and 6 are endorsed by people with higher levels of pandemic fatigue. The item hierarchy indicates that, when people start feeling some pandemic fatigue, they will first feel tired of COVID-19 discussions in the media (item 1). Only respondents with very high levels of pandemic fatigue will acknowledge that they are tired of restraining themselves to save those who are most vulnerable to COVID-19 (item 6). These results support the content validity of the scale.

One item presented a bias by age, with older adults overestimating pandemic fatigue scores in the same construct level. If further research confirms these results, separate item 1 locations may be calculated for each age groups. In the meanwhile, differences by age should be interpreted cautiously.

### Comparison With Prior Work

The known-groups validity results showed that young people reported higher levels of pandemic fatigue. This may be explained by a higher impact of NPI on their social lives, which, in Spain, takes place mostly outside of home. Moreover, young people suffer from asymptomatic or mild disease if infected; they are less likely to adhere to preventive measures [8] and have a decreased risk perception [39]. As a result, they play a crucial role in the increase of incidence rates in several countries, such as in Spain during the summer of 2021 [40]. Younger age was also found to be associated with greater risk of decreasing or diminished interest and avoidance of news about COVID-19 [41]. Therefore, it is necessary to develop campaigns and information strategies specifically addressed to this group of population to overcome these difficulties.

The absence of DIF by sex indicates that the observed significant sex differences are not due to an item bias. We found higher pandemic fatigue scores in women than men, consistent with a study reporting that women are less likely to sustain long-term confinement [42]. However, another study reported higher levels of pandemic fatigue in Spanish men than in women [38]. These discrepancies might be due to how pandemic fatigue was measured in different studies.

In general, our hypotheses about convergent and discriminative validity were supported by the results. As explained in the previous pararaphs, women and young people showed higher levels of pandemic fatigue. Other variables associated with pandemic fatigue were the number and type of preventive behaviors, although with low correlation coefficients. Although pandemic fatigue impacts the adherence to protective behaviors, as stated in the definition by the WHO [4], in Spain, the levels of compliance with the main protective measures (use of face masks, washing hands, and social distance) were very high [13], and the use of face masks was compulsory at the time of data gathering. These results, similar to those in other countries [2], could be an explanation for the low correlation of the CPFS with preventive measures.

Decreased information-seeking behavior is another consequence of pandemic fatigue, and, as our results suggest, people who never or almost never look for information on COVID-19 scored significantly higher on the CPFS. Related to this, items 1 and 3 of the CPFS, which enquire about “information fatigue,” reached the highest mean scores. However, it is difficult to judge if the pandemic fatigue caused the decrease in information-seeking behavior. As hypothesized, people who reported higher levels of pandemic fatigue were those with lower levels of concern, who perceived they are unlikely to be infected, who believed the disease was spreading slowly, who thought they would experience mild disease if infected, or those with depression. Although no causal inferences can be inferred here, other studies have found that less fear of COVID-19 predicted diminished interest in or avoidance of COVID-19 news [41], which is part of the pandemic fatigue definition. In addition, information avoidance predicted a reluctance to engage in COVID-19 preventive behaviors in China [43]. Information avoidance was found to be related to more negative attitudes toward information searching, negative affective responses to risk, and perceived information overload [44].

### Limitations and Strengths

This study has some limitations. First, we used a cross-sectional design, which provides data from the specific time of an evolving pandemic. Second, data were collected using an online survey, which might not reach minority, hard-to-reach population groups. However, the representative sample provides strength for the external validity of the study.

This study presents information on the measurement properties of the CPFS to measure pandemic fatigue in a valid and reliable way. Study strengths include the use of a representative population sample and both CTT and Rasch model methods. Results indicate that the CPFS is useful to monitor the level of population pandemic fatigue from the perspective of individuals. Being formed by only 6 items, the questionnaire is quick to apply, while providing good-quality measurement data for group comparisons. The use of the CPFS could help identify groups of people at risk of higher pandemic fatigue and the design of adequate intervention programs and information campaigns addressed at them.

### Conclusions

In conclusion, the Spanish version of the CPFS is a promising questionnaire to measure pandemic fatigue at the population level, an important public health implication. Its strengths include that it is a brief, unidimensional scale, with a reliability level that allows for group comparisons, absence of bias by gender or education level, and satisfactory validity. As weaknesses and room for improvement, the reliability of the CPFS is not suitable for comparisons at the individual level; 1 item presented bias by age, and there was a lower than expected association with some behavioral aspects.

Further research is needed to test DIF by country. This is very important considering that the CPFS is being used in the WHO behavioral insight survey, in which more than 30 countries are participating. In addition, information on the scale’s sensitivity to change will be very useful to monitor changes due to pandemic progression and public health interventions. In addition, studies of the associated factors to pandemic fatigue in Spain and other countries, measured through the CPFS scale, would be very useful to design public health interventions to prevent and ease pandemic fatigue. Still, our study suggests that younger adults, women, and people with lower risk perception are more susceptible to presenting with higher levels of fatigue. Communication strategies targeted at these groups will likely have a positive impact on lowering pandemic fatigue [18] and, consequently, increase adherence to protection measures.

## Appendix

Multimedia Appendix 1 Description of the variables.

## Abbreviations

CFA: confirmatory factor analyses
CFI: comparative fit index
COSMO: COVID-19 Snapshot Monitoring
CPFS: COVID-19 Pandemic Fatigue Scale
CTT: classical test theory
DIF: differential item functioning
EFA: exploratory factor analyses
NPI: nonpharmaceutical intervention
PSI: person-separation index
RMSEA: root mean square error of approximation
WHO: World Health Organization
