# Supplementary material for: Psychometric Properties of the COVID-19 Pandemic Fatigue Scale: Cross-sectional Online Survey Study
Source: JMIR Public Health Surveill. 2022 Sep 8;8(9):e34675. doi: 10.2196/34675 (PMC9501671; doi:10.2196/34675)
Supplement: Multimedia Appendix 1 [file publichealth_v8i9e34675_app1.docx]

**Supplementary table 1. Description of the variables**

| Variable | Scale or questions | Response options |
| --- | --- | --- |
| Pandemic fatigue | COVID-19 Pandemic Fatigue Scale (CPFS) | 1 (strongly disagree) to 5 (strongly agree) |
| COVID-19 infection | “To your knowledge, are you, or have you been, infected with COVID-19?” | Yes/no |
| Adherence to preventive behaviors | List of 12 measures:  Use of face masks:  1) using face masks following the recommendations  2) wearing face masks in presence of relatives and friends.  Hygienic behavior:  3) ventilating closed spaces;  4) using hydro alcoholic gel or disinfectants;  5) disinfecting surfaces;  6) washing hands;  7) avoiding touching eyes, nose, and mouth with unwashed hands.  Physical distancing:  8) avoiding public transportation;  9) ensuring physical distancing;  10) avoiding social/family events;  11) not visiting relatives and friend if they are in quarantine;  12) avoiding crowded spaces. | 1 (never) to 5 (always) 🡪 recoded to 0 (values 1, 2 and 3) and 1 (values 4 and 5) for analysis |
| Information-seeking behavior | Frequency of looking for information on coronavirus/COVID-19 | 1 (never) to 5 (several times a day) |
| Perceived self-efficacy | “For me, avoiding an infection with coronavirus/COVID-19 in the current situation is..?,” | 1 (very difficult) to 5 (very easy) |
| Level of worry | Level of worry about the coronavirus/COVID-19 in general | 1 (do not worry at all) to 5 (worry a lot) |
| Cognitive risk perception | - Perceived probability of getting infected with coronavirus/COVID-19  - How severe would contracting the coronavirus/COVID-19 be for you | 1 (very unlikely) to 5 (very likely)  1 (not severe) to 5 (very severe) |
| Affective risk perception | “The coronavirus/COVID-19 to me feels…,”:  - Speed of propagation  - Fear  - Mood | 1 (spreading slowly) and 5 (spreading fast)  1 (not fear-inducing) to 5 (fear-inducing)  1 (it does not affect my mood) to 5 (makes me feel depressed) |
